# Achieving beneficial outcomes for children with life-limiting and life-threatening conditions receiving palliative care and their families: A realist review

**DOI:** 10.1177/0269216319870647

**Published:** 2019-08-21

**Authors:** Sarah Mitchell, Karina Bennett, Andrew Morris, Anne-Marie Slowther, Jane Coad, Jeremy Dale

**Affiliations:** 1 Warwick Medical School, University of Warwick, Coventry, UK; 2 School of Health Sciences, University of Nottingham, Nottingham, UK

**Keywords:** Child, palliative care, terminal care, healthcare facilities, manpower and services, realist review

## Abstract

**Background:**

Palliative care for children and young people is a growing global health concern with significant resource implications. Improved understanding of *how* palliative care provides benefits is necessary as the number of children with life-limiting and life-threatening conditions rises.

**Aim:**

The aim is to investigate beneficial outcomes in palliative care from the perspective of children and families and the contexts and hidden mechanisms through which these outcomes can be achieved.

**Design:**

This is a systematic realist review following the RAMESES standards. A protocol has been published in PROSPERO (registration no: CRD42018090646).

**Data sources:**

An iterative literature search was conducted over 2 years (2015–2017). Empirical research and systematic reviews about the experiences of children and families in relation to palliative care were included.

**Results:**

Sixty papers were included. Narrative synthesis and realist analysis led to the proposal of context–mechanism–outcome configurations in four conceptual areas: (1) family adaptation, (2) the child’s situation, (3) relationships with healthcare professionals and (4) access to palliative care services. The presence of two interdependent contexts, the ‘expert’ child and family and established relationships with healthcare professionals, triggers mechanisms, including advocacy and affirmation in decision-making, which lead to important outcomes including an ability to place the emphasis of care on lessening suffering. Important child and family outcomes underpin the delivery of palliative care.

**Conclusion:**

Palliative care is a complex, multifactorial intervention. This review provides in-depth understanding into important contexts in which child and family outcomes can be achieved so that they benefit from palliative care and should inform future service development and practice.

**What is already known about the topic?**

- The population of children and young people with life-limiting and life-threatening conditions and associated palliative care needs is rising internationally.
- Specialist paediatric palliative care services provide benefits for children and their families including symptom control and improved quality of life, a feeling of support and achieving a preferred place of care and death, all of which align with current policy.
- Specialist paediatric palliative care services are inconsistent around the world, and their future development has significant resource implications.

**What this paper adds?**

- This realist review of international literature proposes context–mechanism–outcome configurations which inform a novel programme theory for palliative care for children and young people.
- An increasing number of children live with long-term, complex, life-limiting or life-threatening conditions; established, trusted relationships with healthcare professionals are essential for the delivery of palliative care.
- The importance of these relationships and their potential to trigger underlying mechanisms, including advocacy, affirmation in decision-making and shared emotional impact, are rarely acknowledged in policy.

**Implications for practice, theory or policy**

- Specialist paediatric palliative care services, in countries where they do exist, do not currently have the capacity to provide palliative care to the growing number of children who could benefit from it. Furthermore, the future development of specialist paediatric palliative care services has significant training and resource implications. There is, therefore, a need to consider other possible ways in which to deliver palliative care more consistently. The programme theory proposed in this realist review describes a range of family relevant outcomes that could be considered key to achieving policy and clinical practice outcomes in palliative care. Enabling the healthcare contexts in which underlying mechanisms can be triggered to achieve these child and family relevant outcomes will lead to the more effective delivery of palliative care to children and young people and their families and should be a focus for future policy, service development and commissioning strategies.

## Background

Palliative care is an approach to care which can improve the quality of life for any person living with a life-limiting or life-threatening condition.^1,2^ This review examines the provision of palliative care to children and young people. Age ranges for this population in research vary; for the purposes of this review, children and young people were those aged 0–25 years (hereafter referred to as children).

Over the past 50 years, there have been significant developments in palliative care research and policy, and paediatric palliative medicine has been recognised as a subspecialty of paediatrics in the UK and internationally.^3^ Widely accepted models of children’s palliative care describe three levels of palliative care for children: specialist, core and universal. Core palliative care services are those providing the majority of services and care for children with palliative care needs, including children’s community nursing teams, children’s hospices and paediatricians. Universal services, described as ‘the foundations for palliative care’, include primary care and education.

There has been a significant rise in the number of children living with life-limiting conditions (those which cannot be cured and which will cause premature death) and life-threatening conditions (where curative treatment is possible but may fail).^2,4^ There are almost 400 different conditions in children where palliative care could provide benefit, broadly grouped into four categories (Table 1).^2,5^

**Table 1.** Together for Short Lives categories.^2^

Category	Description
1. Life-threatening conditions for which curative treatment may be feasible but can fail	Access to palliative care services may be necessary when treatment fails or during an acute crisis, irrespective of the duration of threat to life. On reaching long-term remission or following successful curative treatment, there is no longer a need for palliative care services. Examples: cancer, irreversible organ failures of heart, liver, kidney.
2. Conditions where premature death is inevitable	There may be long periods of intensive treatment aimed at prolonging life and allowing participation in normal activities. Examples: cystic fibrosis, Duchenne muscular dystrophy.
3. Progressive conditions without curative treatment options	Treatment is exclusively palliative and may commonly extend over many years. Examples: batten disease, mucopolysaccharidoses.
4. Irreversible but non-progressive conditions causing severe disability, leading to susceptibility to impaired health.	Children can have complex health care needs, a high risk of an unpredictable life-threatening event or episode, health complications and an increased likelihood of premature death. Examples: severe cerebral palsy, multiple disabilities, such as following brain or spinal cord injury.

The involvement of specialist paediatric palliative care services in the care of children and young people with life-limiting and life-threatening conditions is associated with improved symptom control and quality of life for children, their family members feeling more supported, a greater likelihood of care in a place of the family’s choice,^6^ fewer emergency hospital admissions^7^ and fewer intensive care treatments being delivered at the end of life.^8^ However, there are significant inequities in the provision of specialist paediatric palliative care services internationally; specialist services do not have the capacity to manage every child who could benefit from palliative care.^3,9–11^ This, coupled with increasing pressure on other healthcare services which have traditionally played a key role in the delivery of a palliative care, such as community nursing and primary care,^12^ is leading to marked inconsistencies in how children and their families experience such care. Outcomes described as important in policy, including advance care planning and discussions about a preferred place of death, are not consistently offered to children with life-limiting and life-threatening conditions and their families.^13,14^

### Aims

The aim of this realist review is to describe when and how palliative care provides benefits, from a child and family perspective. The realist approach allows the description of context–mechanism–outcome configurations (CMOCs) and a programme theory to provide insights into how palliative care could be delivered more broadly to children and families.

### Rationale for a realist review

A realist review is a theory-driven, explanatory, systematic approach which aims to investigate how, when, for whom and to what extent a particular intervention (or ‘programme’) works.^15,16^ A realist review of the evidence relating to paediatric palliative care has the advantage over other review methods in that it allows for consideration of palliative care as a broad and complex intervention. It takes into account the fact that palliative care requires the active input of individuals, specialists and non-specialists, who are embedded in social infrastructures, such as hospitals and community services, and whose role is influenced by others, including patients and colleagues. The impact of institutional and system factors, such as local and national policy guidance and commissioning, provides further complexity.

The goal of a realist review is to explain the contexts (C) in which hidden underlying mechanisms (M) are triggered in order to generate outcomes (O) of interest. CMOCs are proposed and used to develop a programme theory that is ‘useful’, ‘testable’ and policy relevant^16^ (Table 2).

**Table 2.** Glossary of realist terms.

Term	Explanation
Context	Pre-existing structures, settings, environments, circumstances or conditions that influence whether certain behavioural and emotional responses (i.e. mechanisms) are triggered.
Context–mechanism–outcome configurations (CMOCs)	Describe the causal relationships between contexts, mechanisms and outcomes, that is, how certain outcomes are achieved through mechanisms being triggered in certain contexts.
Mechanisms	The behavioural or emotional response which is triggered in certain contexts. Mechanisms are context sensitive and are usually hidden.
Outcome	The impact of mechanisms being triggered in certain contexts.
Programme theory	A set of theoretical explanations about how a particular programme, process or intervention is expected to work.
Mid-range theory	Theoretical explanations which are suitable for testing through further research. A programme theory can be specified at the mid-range.

Source: Adapted from Papoutsi et al.^17^

## Methods

The review was conceptualised in August 2015 and carried out over 2 years. Ethical approval was not required. A protocol has been published in PROSPERO (registration no: CRD42018090646 https://www.crd.york.ac.uk/prospero/display_record.php?RecordID=90646).

The review followed the RAMESES standards.^18^ (1) An initial programme theory was identified, and the purpose of the review was clarified. (2) This was followed by a detailed iterative search for research evidence. (3) Articles were selected for inclusion based on their relevance to the research questions. (4) Relevant data were extracted and organised into a Word table. (5) The final stage of the review was data synthesis, developing CMOCs and a testable, mid-range programme theory.^16,18^

### Step 1: identification of an initial programme theory and clarification of the scope of the review

Our initial programme theory, that palliative care for children ‘works’, was informed by our systematic review ‘Specialist paediatric palliative care services: what are the benefits?’.^6^ The review described an association between the involvement of specialist paediatric palliative care in a child’s care and improved quality of life, including symptom control, a feeling of support for families, increased likelihood of achieving a preferred place of care and death. The review did not investigate how, when or why these outcomes were achieved.

A scoping review was conducted, comprising an exploratory Internet-based literature search, review of policy documents, collection of relevant articles via social media and at conferences (Table 3) and regular discussion with a stakeholder group of professionals and parents (the West Midlands Paediatric Palliative Care Network) who met every 3 months through the course of the review. This revealed a diverse range of literature in paediatric palliative care, with articles focussing on many different aspects of care including the child and family experience, symptom control, advance care planning, organ donation, complementary therapies, spirituality and the perceptions of healthcare professionals. Following discussion with the stakeholder group and research team, a decision was made to focus on the experiences of children and their families in relation to palliative care, prioritising research that provided insights into their experiences and perceptions, rather than the experiences of professionals. The research questions that emerged were as follows:

1. What are the beneficial outcomes (O) described by children with life-limiting and life-threatening conditions and their families in relation to palliative care?
2. What are the contexts (C) that determine whether or not these mechanisms produce the outcomes?
3. What are the mechanisms (M) triggered in these contexts to produce beneficial outcomes for children and families?
4. What are the implications for future research, policy and practice?

**Table 3.** Sources of information to identify existing theories.

Area of initial search	Sources
Internet	Google, Google Scholar, NHS, voluntary sector and government websites and the Cochrane library
Desk drawer search	Articles already known to the researchers Search of key textbooks
Social media	Saving relevant articles found through Twitter, Facebook and Together for Short Lives newsletters
Conferences	Posters and presentations and abstracts Reflective notes
Stakeholders	West Midlands Paediatric Palliative Care Network meetings Reflective notes

NHS: National Health Service.

### Step 2: systematic literature search

A formal database search was designed by S.M. with support from the specialist librarian at the University of Warwick. The search was carried out in November 2015. Broad search terms were tested in PubMed (Palliat* AND Paediatr*/ Pediatr*); searches were then carried out in Allied and Complementary Medicine Database (AMED), Applied Social Sciences Index and Abstracts (ASSIA), Cumulative Index to Nursing and Allied Health Literature (CINAHL), Embase, PsycINFO, Web of Science and Education Resources Information Center (ERIC), with the search terms modified and adapted for each database, but kept deliberately broad (detailed in Table 5). Forward and backward citation tracking was conducted. The database search was of papers published since 1980, but articles were not excluded based on the date of publication. The search was limited to papers published in English. Relevant references were collected over 2 years via citation alerts and social media and at conferences, and the database search was repeated in December 2017. The aim was to gather evidence to refine and test the initial programme theory, rather than to conduct an exhaustive search of the paediatric palliative care literature.

### Step 3: document screening and selection

References were exported to citation management software (EndNote) and screened for duplicates. S.M. reviewed all the titles and abstracts in chronological order, to gain an understanding of shifts and changes in the literature over time, and grouped the articles into categories according to the subject and focus of the research. Inclusion and exclusion criteria were devised (Table 4). Articles that provided empirical research evidence or family accounts about the experiences of children and families in relation to palliative care were included and retrieved as full texts (by S.M.). Expert professional opinion articles, practice reviews and editorials were deliberately excluded.

**Table 4.** Inclusion and exclusion criteria.

Inclusion	• Empirical research or systematic reviews about the experiences of children and families in relation to the delivery of palliative care (either specialist paediatric palliative care services (those supported by a consultant in Palliative Medicine), other paediatric palliative care services or any important aspect of palliative care such as communication). • Children and/or families are the research participants • Children are defined as aged 0–25 years (palliative care services and research studies vary in their age thresholds) • Children with life-limiting or life-threatening conditions (as defined by Together for Short Lives)2
Exclusion	• Opinion pieces, editorials and practice reviews • Research about the opinions and experiences of healthcare professionals • Neonatal/antenatal/adult palliative care

### Step 4: extracting and organising data

The review team (S.M., K.B. and A.M.) read and re-read the articles and met regularly to consider the trustworthiness and rigour of those that were included. Article characteristics (citation, year, country, type of paper, aims, methods and participants) were summarised in a Word data extraction table (Supplemental Appendix 1) by K.B. and A.M., with regular discussion and consistency checking with S.M. and the research team. Relevant sections of text were coded by S.M. and K.B. through a process of manual annotation and in NVivo. An inductive approach was taken, with codes and concepts originating from the data, using the following questions to guide the process:^19^

1. What does this section of text describe about the important factors in relation to palliative care for the child and family?
2. Is the section of text referring to context, mechanism or outcome?

A second data extraction table was used to document key relevant sections of text that were used to inform interpretations about what was functioning as context, mechanism or outcome within CMOCs (Supplemental Appendix 2).

### Step 5: data analysis and synthesis

The aim of the data analysis was to interpret and explain the ‘hidden’ mechanisms, triggered in certain contexts, leading to beneficial outcomes for children and families. The coded sections of text were used to develop CMOCs, using the following questions as a guide:

1. What is the context? What outcomes are described? What are the hidden mechanisms? What is the CMOC?
2. How does the CMOC relate to patient and family experience?
3. Is the evidence trustworthy and rigorous?

The analysis was conducted by S.M. and K.B. Consistency and accuracy were checked, and potential CMOCs were debated, compared and consolidated by the research team (S.M., J.D. and A.-M.S.). Analytical strategies were employed including juxtaposition of data sources (aligning evidence to inform and clarify a theory), exploration and reconciliation of discrepancies in the data and adjudication of data quality.^18,20^ Where further evidence was required to adjudicate an argument, S.M. conducted a purposive search in the organised data set from the wider literature search (stored in EndNote). An explanation of how this review fulfils the RAMESES quality standards for a realist review is provided in Supplemental Appendix 3.

## Search results

A total of 5930 articles were identified from the database search (Table 5). Fifty-five further articles were identified through desk drawer searching, forward and backward citation searching and the collection of articles from social media. After title and abstract screening, 5211 articles were excluded as they were either not relevant to the research questions or duplicates. According to the focus of the research, 774 articles were grouped into broad conceptual categories. 714 articles were editorials, opinion pieces, practice reviews or research that did not include children or families as participants. Sixty articles that met the inclusion criteria (children and families as the research participants) comprised the final data set. The children included in the studies had a diverse range of life-limiting and life-threatening conditions.

**Figure 1.**
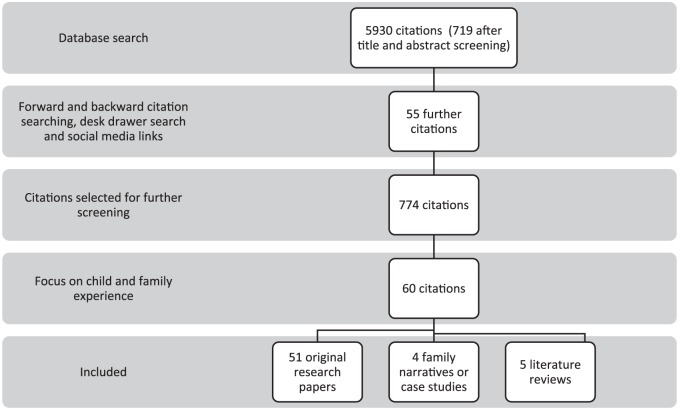
PRISMA flow diagram.

The characteristics of the included studies are provided in Supplemental Appendix 1. The data screening and extraction processes are shown in the Preferred Reporting Items for Systematic Reviews and Meta-Analyses (PRISMA) flow diagram (Figure 1).^21^

**Table 5.** Literature search.

Database	Search terms	Articles found on searching	Articles selected after title and abstract screening
AMED	Palliat* AND (Paediatr* or Child*)	721	209
ASSIA	Palliat* AND (Paediatr* or Child*)	643	29
CINAHL	Palliative care AND paediatric/children	168	41
Embase	Palliat* AND Paediatr*	1041	140
PsycINFO	Palliat* AND Paediatr*	69	28
PubMed	Palliat* AND Paediatr*/Pediatr*	1805	181
Web of Science	Palliat* AND Paediatr*	1339	89
ERIC	Palliative care AND Paediatric/children	144	2
Desk drawer search	N/A	55	55
Total		5930	719

AMED: Allied and Complementary Medicine Database; ASSIA: Applied Social Sciences Index and Abstracts; CINAHL: Cumulative Index to Nursing and Allied Health Literature; ERIC: Education Resources Information Center; N/A: not applicable.

51 were original research papers, five were literature reviews,^22–26^ two were first-person family narratives,^27,28^ one was a case study^29^ and one was an analysis of a diary.^30^ 22 studies included children with an oncology diagnosis,^22,23,26,27,29–46^ five concerned those with non-malignant disease,^28,47–50^ and 33 included both.^8,24,25,51–80^

Of the 51 research studies, 15 were carried out with parents,^35,39,40,43,47,55–57,60,61,65,70,75,77,79^ one with parents and grandparents^48^ and four with both children and parents.^53,59,62,76^ Three studies included only children as participants: a retrospective cohort population study,^8^ a qualitative interview study where children were interviewed alone^45^ and a longitudinal observational study.^49^ Two studies included siblings.^67,72^ 26 studies were carried out with parents post-bereavement. ^31–34,36–38,41,42,44,46,50–52,54,58,63,64,66,68,71,73,74,78,80,81^

Studies were heterogeneous in terms of methods; the majority made use of qualitative methods including individual interviews,^31,32,34–37,39,42–47,51–56,58,62,63,66–68,70,75,76,78,79^ focus group interviews^38,41,64,75,76^ or written questionnaires.^33,50,62,71–73,77,81^ Several studies conducted quantitative analysis on questionnaire findings.^32,57,60,65,66,74^ The studies represented an international evidence base, with studies from the UK,^27,28,38,46,59,62,63,65,75,76^ Australia,^36,37,55,71,79^ Canada,^8,39,40,42,47,48^ the USA,^23,24,26,29,31,32,49–54,56–58,60,61,64,66,68,70,77,81^ Germany,^34,41^ Holland^43,73,74^ India,^35^ Ireland,^22^ Malaysia,^78^ New Zealand,^67,72^ Sweden,^25,33,44,82^ Switzerland^80^ and Turkey.^30^

## Findings

### Overview of review findings

The findings provide insights into and understanding of the beneficial outcomes described by children with life-limiting and life-threatening conditions and their families in relation to palliative care and when and how these are achieved. They are divided into four conceptual areas: (1) family adaptation and experiences, (2) the child’s situation, (3) relationships with healthcare professionals and (4) access to palliative care services. A narrative is provided for each area, followed by realist analysis and CMOCs. A programme theory, derived from the CMOCs, is then presented.

#### Family adaptation and experiences

A child becoming seriously unwell or dying alters family life in ways which parents and siblings cannot anticipate or prepare for.^35,48,56,67^ Parents grieve for the loss of the child’s health, struggle with a feeling of responsibility for their child’s wellbeing and have to adjust their hopes and expectations of parenthood and the future.^33,35,56,60,75^ The diagnosis of a condition such as cancer brings an immediate realisation of the precariousness of life,^48^ whereas parents of children with non-malignant, congenital conditions describe a more gradual realisation, with the severity of the child’s condition being underemphasised by healthcare professionals who are ‘too considerate’.^55^

Families adapt over time, carrying out essential practical tasks^65,76^ and becoming experts in both their child’s condition and the impact it has on their family.^22,27,61,76^ They find new meaning and purpose in their lives,^44,48,61^ adopting the role of a carer, spending more time in hospital and leaving work, which can lead to feelings of vulnerability, isolation, fatigue, depression and anxiety and a perception that no one understands the family’s burdens.^60,61,79^ Support is drawn from a wide variety of sources, including other parents of children with the same condition, friends and the local community.^44,47,60,73^

Life with intensive medical treatments and chronic uncertainty becomes normal,^29,48,50,63^ and the parent–child bond develops in the context of an illness that is often characterised by unexpected crises and ‘moments of realisation’ when the threat to the child’s life is recognised.^51,59,60,79^ Coping with this normality is challenging and stressful.^65,77^ Parents adopt a number of strategies such as trying to maintain hope and ‘staying positive’.^38,39,45,48,51^ Parents and families describe a need to be respected as experts in their child’s condition, to be involved in care decisions, and for their beliefs and opinions to be taken seriously at times when their child is critically unwell and may die,^24,40,54,57,58,70,72,74^ but this does not always happen in practice.^66^ As ‘protectors’ of the child,^79^ parents are caught between conflicting emotions, neither wanting their child to suffer nor wanting their child to die,^44^ but they may not have to fully acknowledge that their child is dying in order to be willing to place the emphasis of care on lessening of suffering.^31^ When difficult decisions are to be made, affirmation in their decision-making from a healthcare professional who has witnessed the magnitude of the task is valued.^71^

Parents can experience disempowerment related to the healthcare environment in which their child is receiving care. The intensive care unit has been described as ‘bewildering’,^68^ and parents have described feeling unable to raise concerns about their child’s care if they feel grateful to a service or perceive that by virtue of being in a specialist centre, their care is the best it can be.^32,51,62^ Clinical concerns, including symptoms, have been found to be underreported by healthcare professionals compared to parents who may not always feel able to raise their concerns.^32,33,47^

Studies suggest that healthcare professionals recognise that a child is dying before family members do.^31,32^ This may happen very late in the course of illness, sometimes not until death is imminent.^50^ Parents describe receiving the news that their child is going to die as ‘a crushing, stunning defeat after a prolonged and painful struggle’,^27^ like ‘gripping my heart and squeezing’^46^ and ‘like being covered in a wet and dark blanket’.^44^ They may have difficulties understanding and assimilating information about the incurability of their child’s condition,^44^ perhaps because this represents a significant change from a cure-focussed management plan, particularly when the underlying condition is cancer. Some parents are never explicitly told that their child is dying.^66^ It is important to note that family narratives and case studies suggest that family members are aware of the possibility of the death of the child throughout the course of illness.^28,29,46,51,59^

### Realist analysis

**Figure 2.**
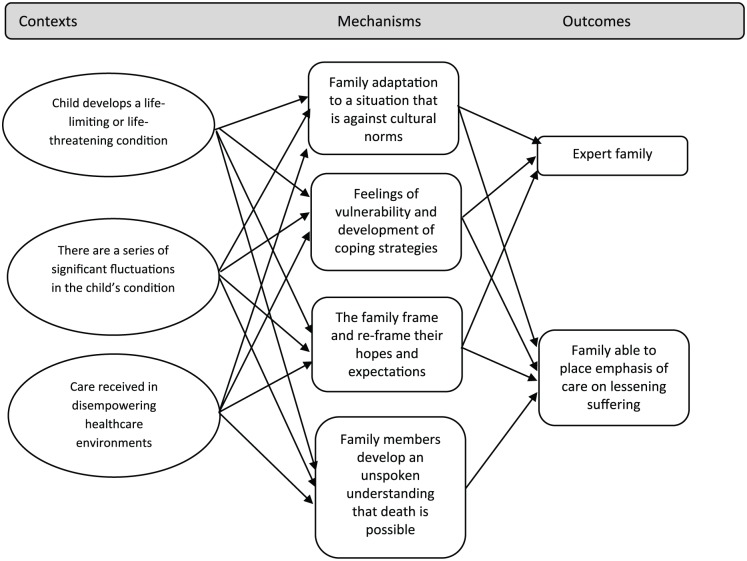
CMOCs relating to family adaptation.

There is much to learn from the literature about the experience of families with children who have life-limiting or life-threatening conditions. Descriptions of their experiences highlight important contexts for the delivery of palliative care, as both a broad approach and as a specialist service. These contexts include the fragility of the child’s condition and chronic uncertainty. Mechanisms triggered in these family contexts include adaptation to a situation that is against cultural norms, continually adjusting expectations for family life and developing coping strategies (mechanisms). Family members frame and re-frame their hopes and expectations (mechanism) and develop significant expertise in the management and impact of child’s condition (outcome). Families are disempowered and intensely vulnerable in their situation, both in terms of the uncertainty that they live with and in their interactions with healthcare environments and systems (context). They have an awareness that their child may die, but this may remain unspoken until late in the child’s illness (mechanism). However, this awareness may allow them to place the emphasis of care on lessening suffering (outcome), even if the possibility of dying remains unspoken. These CMOCs are outlined in Figure 2 below:

#### The child’s situation

The ability of children with life-limiting and life-threatening conditions to take part in conversations about their healthcare varies according to their age, developmental stage, psychological and cognitive factors related to their condition and the behaviour of the adults around them.^66^ Parents are often the surrogate decision makers, with children becoming passive recipients of the decisions that are made for them,^32^ a situation in which they display both resilience and dignity.^27^

In the few studies where children participated, they expressed a desire to live their lives as normally as possible despite their abnormal circumstances.^38,76^ Their priorities included seeing friends and attending school.^62^ They wished to receive truthful information, in a way that they could understand and at the same time as their parents.^45^

Parents worry about a right or wrong way to discuss death and dying with their children.^27,67^ Cultural beliefs, a desire to protect the child or a perception that their child is ambivalent about taking part in healthcare discussions lead parents to consider conversations with their child about the possibility of death to be inappropriate or unacceptable.^35,64,67^ Even without conversations, parents describe seeing their child’s understanding of their situation change over time,^29^ as they develop a ‘tacit understanding’ that they may die. Some parents and caregivers feel that explicit conversations about dying become unnecessary because the child already understands the reality of their situation.^55,67^

### Realist analysis

**Figure 3.**
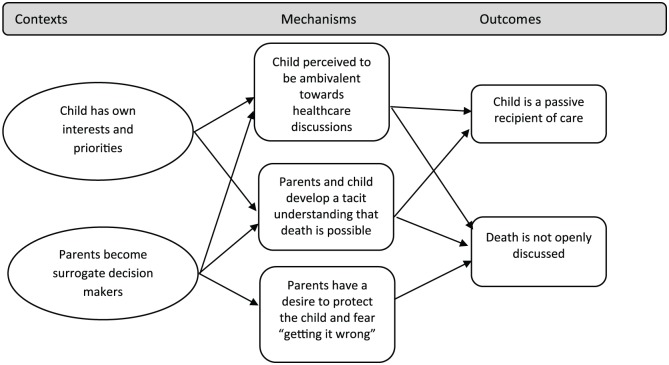
CMOCs related to the child’s situation.

Figure 3 outlines CMOCs related to the child’s situation. Children express their own interests and priorities for life (context); parents are often their surrogate healthcare decision makers (context). Children may be ambivalent about decisions related to their health, or may be protected by their parents, therefore becoming passive recipients of the care decisions that are made for them (outcome). The possibility of dying may not be openly discussed (outcome), but a tacit understanding that the condition may lead to death has been described among children (mechanism).

#### Relationships with healthcare professionals

The relationships that develop between children, family members and healthcare professionals are critical to the family experience.^23,51,63^ Families describe the necessity of authentic relationships and want to feel that healthcare professionals are experienced, competent and can be trusted.^55,68^ Open, honest communication, care co-ordination, accessibility and availability are valued.^37,42,51,53,64,66,77,80^ Children and parents appreciate healthcare professionals who take the time to get to know the child, even to the extent of ‘developing a friendship’.^53^ The individualised and intimate knowledge of the family situation which underpins these relationships is often achieved through continuity of care.^62,76^ It may be one specific healthcare professional who advocates for the family and is perceived to be particularly helpful.^74,76^

Families value the emotional investment made by some healthcare professionals, demonstrated through compassion and acts such as appearing to care for the child as ‘one of their own’, attendance at a memorial service or making contact in bereavement.^52,64,68^ Being with families at their most vulnerable time requires understanding of the physical and psychological distress that they might be experiencing and an ability to bear this with them, a situation which can lead healthcare professionals to experience their own feelings of distress.^56,66,69^

Conversely, relationships that are perceived as ‘poor’ by parents carry significant risks of harm. A single event, such as the insensitive delivery of bad news, parents feeling patronised or dismissed or that their judgement is disregarded, can lead to lasting distress.^29,33,47,51,53,68^ A lack of continuity leading to different healthcare professionals asking the same questions several times can be ‘disturbing’.^25^ Times when parents feel the opinions of healthcare professionals have been ‘inflicted’ upon them, or when their individual needs have been subsumed to standard procedures rather than being listened to, may lead to significant conflict.^68^

### Realist analysis

**Figure 4.**
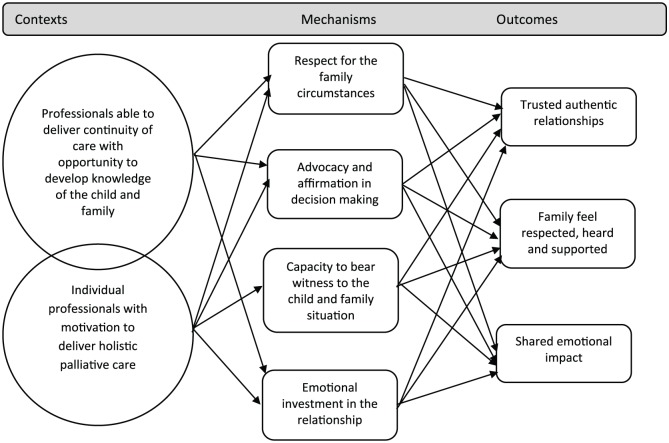
CMOCs outlining relationships with healthcare professionals.

There are two important interdependent contexts for healthcare professionals, which trigger mechanisms leading to beneficial outcomes for children and families. Individual professionals differ in their approach, with some more motivated to deliver a holistic approach to care (context). Continuity of care allows the development of detailed knowledge of the child and family situation over time (context). Mechanisms triggered in these contexts include respect for the family circumstances, advocacy and affirmation in decision-making, personal emotional investment and a capacity in the healthcare professional to bear witness to the family situation. These mechanisms lead to outcomes including trusted, authentic relationships between children, their families and healthcare professionals in which children and families feel respected, heard and supported. They feel that the healthcare professional shares the emotional impact of the child’s condition (outcome). These CMOCs are outlined in Figure 4 below:

#### Access to palliative care services

When available, specialist paediatric palliative care services are associated with a range of benefits including a feeling of support for families and improved symptom control.^8,32,34,65,78,81,83^ Symptom control can be particularly challenging, given each child’s individual condition and circumstances.^47,49,66^

However, barriers to referral exist, including variable perceptions and opinions of the term ‘palliative care’ among professionals, children and their families.^84^ Research suggests that family members view ‘palliative care’ as a distinct phase at the end of a child’s life, ‘the beginning of the end’. They fear it as a point at which they will lose contact with the healthcare services they know, a situation that can be ‘terrifying’.^75^

Parents who receive care from specialist paediatric palliative care services report that they wish they had been introduced to these services earlier in the course of the child’s illness.^55,62^ They are more likely to accept a referral once they have been provided with detailed information which addresses their own preconceptions of ‘palliative care’.^84^

Children’s perceptions of palliative care services are largely unknown. They have been found to be reluctant to accept new services or healthcare providers who are introduced towards the end of life.^64^ However, bereaved parents are more likely to describe their children as calm and peaceful during the last month of life if they have had contact with a hospice.^32^

### Realist analysis

**Figure 5.**
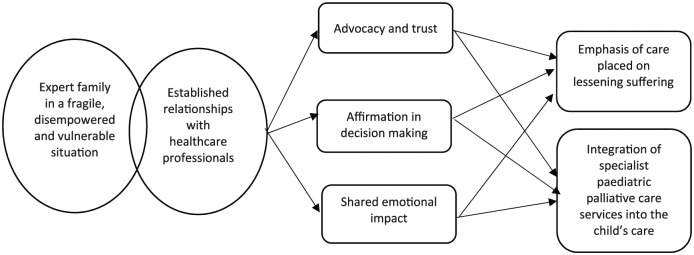
CMOCs related to palliative and end-of-life care.

The analysis so far highlights the intense vulnerability of families who are experts in the care of the child and their condition, when they realise that their child may die (context). The relationships with trusted healthcare professionals that have been established through the course of the child’s illness are key and function as a context for the delivery of palliative care, including being able to place an emphasis of care on lessening suffering and making a referral to specialist paediatric palliative care services (outcomes). These are important precursors to being able to consider policy-relevant outcomes in the care of individual children and their families, such as advance care planning, and to ensuring they have access to specialist palliative care expertise and services, such as children’s hospice support. Negative perceptions of palliative care and challenges with introducing new professionals or services late in the course of the child’s illness can make difficult the introduction of specialist services as the child approaches the end of life. The underlying mechanisms, including advocacy, trust and affirmation in decision-making, can all help with this process (Figure 5).

#### Development of a programme theory

The realist analysis related to the delivery of palliative care service and policy outcomes starts by taking the outcomes described through the formulation of CMOCs related to the family experience and the child’s situation, as important contexts for the delivery of palliative care, the children with their own interests and priorities and the expert family who are disempowered and vulnerable in their situation, both of whom may have an unspoken awareness that death is possible.

**Figure 6.**
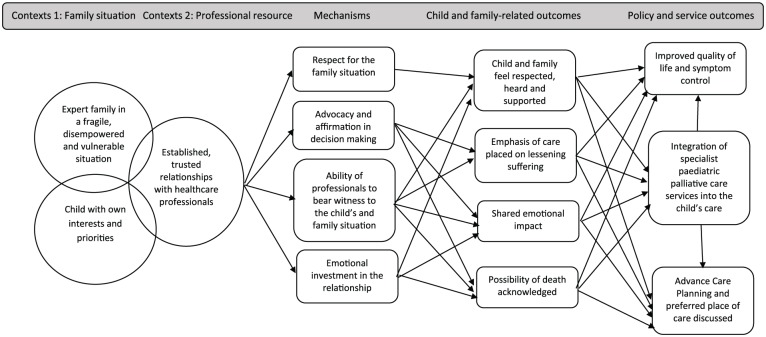
Proposed programme theory.

Important child and family-related outcomes are feeling respected, heard and supported and being able to place emphasis on lessening the child’s suffering. These depend on established, trusted relationships with healthcare professionals who are motivated to deliver a palliative care approach and can provide continuity of care through the course of the child’s illness. Relationships of this nature can be considered as being a professional resource context for the delivery of palliative care. The mechanisms which underpin these relationships are key and include respect for the family circumstances, advocacy, affirmation, an ability in the healthcare professional to bear witness to the child and family situation and emotional investment in the relationship. Through these relationships, shared emotional impact (outcome) and open acknowledgement of the fragility of the child’s condition and the possibility of dying (outcome) could be achieved. These are key precursors to conversations during which child and family preferences and priorities, and referral to specialist paediatric palliative care services, can be discussed (outcomes). Achieving these outcomes supports more consistent delivery of the service outcomes identified in our systematic review, including improved quality of life and symptom control and a feeling of support for families. Policy outcomes, including achieving a preferred place of death, may also be more likely to be achieved (Figure 6).

## Discussion

### Summary of findings

This review has led to the development of a programme theory that proposes how the delivery of palliative care to children and their families could be improved, through a series of explanatory mechanisms, triggered in certain contexts, to produce outcomes described as important to families. The programme theory brings together the contexts, mechanisms and outcomes from the literature and relates these to desired policy and palliative care service outcomes.

The review has provided insights into the highly individual and unique knowledge that families develop about the management of their child with an, often complex, life-limiting or life-threatening condition. Their hopes and expectations are shaped by constant adaptation to uncertainty and a sometimes unspoken awareness that the child may die. These child and family circumstances are contexts in which palliative care must be delivered. Where established, trusted relationships with healthcare professionals exist, mechanisms, including advocacy and emotional investment in the relationship, are triggered, which lead to child and family outcomes including feeling respected, heard and supported, and that their emotional burden is shared. These child and family outcomes may lead to a more open acknowledgement of the possibility of death and the ability to place the emphasis of care on lessening suffering.

### Strengths and limitations

The strength of the realist approach is its explanatory nature. This review set out to investigate what works for children and families, when, how and in what circumstances in terms of palliative care, and the iterative search strategy reduced the risk of missing major concepts that are relevant to the delivery of a palliative care approach to children and families. The evidence was international and included a diverse range of clinical conditions, adding to the applicability of the findings across healthcare systems. There was a lack of research where children were participants, and more work needs to be done to understand the potential benefits of palliative care from their perspective.

The majority of the studies reviewed were qualitative, and a strength of the review is that this allowed relevant contexts, mechanisms and outcomes to be abstracted from rich, in-depth data. Expert opinion articles, such as editorials and practice reviews, were deliberately excluded. Evidence related to the child and family experience was prioritised, in order to understand experiences from their perspective. The rationale for this was that, given the paucity of research evidence in the field of paediatric palliative care, much current policy to date has been informed by expert opinion. Research that investigates whether and how current policy aligns with the child and family experience is vital. There is a paucity of published research in this field, particularly research relating to the child’s experience. Most of the studies included bereaved parents as the participants, with varying lengths of time since their bereavement, another possible limitation. Recollections of experiences can change over time,^85^ and there may be participant bias in these studies, with those who can cope or who are more motivated to improve palliative and end-of-life care for children being most likely to participate.

### What this study adds

This realist review addresses an important gap in the evidence, providing an understanding of the contexts that are required in order to achieve beneficial outcomes for children with palliative care needs and their families. The insights are valuable, given the challenge of translating the words of policy into clinical practice. The programme theory proposes there are important child and family outcomes, which may underpin the delivery of wider policy goals and palliative care service outcomes.

### Recommendations for research, practice and policy

In order for policy goals and standards to be achieved in paediatric palliative care, organisational policy and intervention strategies should be developed that recognise the key importance of family relationships with healthcare professionals. Enabling the contexts that trigger mechanisms leading to important child and family outcomes could result in a palliative care approach being delivered more consistently. Intervention strategies include providing support for those who are motivated to provide palliative care, as well as accessible education and training opportunities. It also requires healthcare leaders and those involved in service design to value continuity of care and to enable time resource for key interpersonal relationships to develop.

Paediatricians are frequently involved in the care of children with life-limiting and life-threatening conditions, and the care of children who die, from early on in their career.^86,87^ There is wide variation in the confidence levels of paediatricians in terms of the delivery of palliative care,^88^ and mixed levels of willingness to undertake further training,^88,89^ perhaps because palliative care is poorly understood. Accessible and relevant training and education opportunities need to be developed, including increasing awareness and changing attitudes around what palliative care is and education about the role of specialist services, where they are available.

The presence of role models, such as members of a specialist paediatric palliative care team, can have a positive impact in terms of increasing understanding of palliative care.^90^ Further research to understand how healthcare professionals develop the professional values and behaviours that make the delivery of palliative care possible, including whether there is a ‘type’ of healthcare professional or family that are more likely to engage with palliative care, would be valuable.^8^

The provision of clear and comprehensive information to families outlining available professionals and services, including specialist paediatric palliative care services, early on in the course of the child’s condition could potentially be helpful. Currently, they may receive information about available services through informal peer support networks, including via social media. One important consideration for the future will be understanding the preferred information sources of children and their families and their needs and preferences regarding that information. This is one area for future investigation. To date, there has been very little research that investigates the experiences of children with life-limiting and life-threatening conditions in relation to their experiences of healthcare services, which is also an important area for future work.

## Conclusion

In conclusion, this review has described how outcomes that are important to children and families, including feeling heard, respected and that their emotional burden is shared, underpin their experience of palliative care. These outcomes are achieved through the development of established, trusted relationships with healthcare professionals and hidden mechanisms triggered within these relationships including advocacy and affirmation in decision-making. Motivation to deliver palliative care and an ability to bear witness to the child and family situation are necessary within healthcare professionals. These are nuanced and hidden influences, which are rarely acknowledged in policy, but require more attention, since they lead to child and family outcomes that underpin beneficial policy and service outcomes in palliative care.

## Supplemental Material

870647_supp_mat – Supplemental material for Achieving beneficial outcomes for children with life-limiting and life-threatening conditions receiving palliative care and their families: A realist review Click here for additional data file. Supplemental material, 870647_supp_mat for Achieving beneficial outcomes for children with life-limiting and life-threatening conditions receiving palliative care and their families: A realist review by Sarah Mitchell, Karina Bennett, Andrew Morris, Anne-Marie Slowther, Jane Coad and Jeremy Dale in Palliative Medicine
