# Supplementary material for: Achieving beneficial outcomes for children with life-limiting and life-threatening conditions receiving palliative care and their families: A realist review
Source: Palliat Med. 2019 Aug 21;34(3):387–402. doi: 10.1177/0269216319870647 (PMC7074600; doi:10.1177/0269216319870647)
Supplement: 870647_supp_mat – Supplemental material for Achieving beneficial outcomes for children with life-limiting and life-threatening conditions receiving palliative care and their families: A realist review [file 870647_supp_mat.pdf]

Appendix 1 Quality Standards (table adapted from RAMESES standards and Papoutsi et al (16)

Quality criteria	How the criteria were fulfilled
The research topic is appropriate for a realist review	Palliative care is a complex intervention that require the active input of individuals, whose role is influenced by other individuals including patients and colleagues. Palliative care services are embedded in other social infrastructures (such as hospitals, hospices and primary care) and affected by institutional and system factors (such as local and national policy guidance and commissioning).<br>One of the aims of the review is to produce policy relevant recommendations, which is one of the specific aims of realist review.
The research question is constructed in such a way as to be suitable for realist analysis, and is sufficiently and appropriately focused	The research questions broadly ask "when" and "how" palliative care provides benefit to children and their families. This was refined further to specifically ask about the mechanisms by which palliative care provides benefit, and the contexts in which these mechanisms are triggered.
The review demonstrates understanding and application of a realist philosophy and realist logic that underpins a realist analysis	The review followed Pawson's five stages of realist review, and the RAMESES standards. A realist logic of analysis allowed for contexts, mechanisms and outcomes to be identified in the data, with a focus on generative causation and the subsequent development of CMOCs.
An initial realist programme theory is identified and developed	The initial programme theory was derived from policy documents and a systematic review. This was refined and developed through engagement with stakeholders
The search process is such that it would identify data to enable the programme theory to be developed, refined and tested	The search strategy was deliberately broad and extensive, including multiple data sources. Literature searching took place over two years
The selection and appraisal process ensures that sources relevant to the view containing material of sufficient rigour are identified.	A decision was made to include empirical research evidence related to paediatric palliative care, rather than opinion pieces or editorials, to ensure that the included evidence was rigorous. Rich, in-depth data was yielded via the search strategy with data identified to configure CMOs.
The data extraction process captures the necessary data to enable a realist review	An iterative process of data coding and extraction took place, with relevant data coded and captured to support specific CMOCs.
The realist synthesis is reported using the items listed in the RAMESES reporting standard for realist syntheses.	The paper has followed the reporting standards.

For Peer Review

For Peer Review

PALLIATIVE MEDICINE AUTHOR SUBMISSION CHECKLIST: When and how does palliative care "work" for children with life-limiting and life-threatening conditions and their families? A realist review. Dr S Mitchell

Please complete this checklist for all papers submitted. Please indicate, very briefly, how this has been addressed. This checklist is a mandatory upload on submission.

Item	Explanation	How this has been addressed (briefly, a sentence will suffice)
Article title	***WHY: Because we want readers to find your work.*** Have you followed our guidelines on writing a good title that will be found by search engines? (E.g. with methods in the title, use of common words for the issue addressed, no country names, and possibly indicating findings). If your study has an acronym is it included in the title?	Title includes terms palliative, children and realist review
Abstract	***WHY: Because structured abstracts have more detail for readers and search engines.*** Have you followed our guidelines on writing your structured abstract? Please remember we have separate abstract structures for original research, reviews and case reports. There should be **no** abbreviations in the abstract, EXCEPT a study acronym which should be included if you have one. If a trial (or other design formally registered with a database) have you included your registration details?	Guidelines for review abstract followed. Includes link to PROSPERO registered protocol
Key statements	***WHY: Because readers want to understand your paper quickly.*** Have you included our key statements within the body of your paper (after abstract and before the main text is a good place!) and followed our guidelines for how these are to be written? There are three main headings required, and each may have 1-3 separate **bullet** points. Please use clear, succinct, single sentence separate bullet points rather than complex or multiple sentences.	Key statements included
Keywords	***WHY: Because MeSH headings mean it is properly indexed.*** Have you given keywords for your study? We ask that these are current **MeSH** headings unless there is no suitable heading for use (please give explanation in cover letter). https://meshb.nlm.nih.gov/search	MeSH keywords included
International relevance	***WHY: We have readers from around the world who are interested in your work.*** Have you contextualised your work for an international audience and explained how your work contributes to an international knowledge base? Avoid drawing from policy from one context only, think	Review draws on international literature and addresses an internationally relevant concern

For Peer Review

	how your work could be relevant more widely. Do define terms clearly e.g. hospice has a different meaning in many countries.	
Publishing guidelines	***WHY: Because clear and robust reporting helps people interpret your work accurately***<br>Have you submitted a completed checklist for a relevant publishing guideline as a supplementary file? http://www.equator-network.org/ These include CONSORT, PRISMA, COREQ checklists, but others may be more relevant for your type of manuscript. If no published checklist exists please create one as a table from the list of requirements in your chosen guideline. If your study design does not have a relevant publishing guideline please review closest matches and use the most appropriate with an explanation.	Quality assessment with RAMESES standards included in main text. PRISMA checklist submitted.
Word count	***WHY: Because readers want to find the core information quickly.***<br>Does your paper adhere to our word count for your article type? Please insert number of words in the box to the right. Remember that tables, figures, qualitative data extracts and references are not included in the word count.	4999
Figures and tables and/or quotations	***WHY: Because readers want to find the core information quickly.***<br>Have you adhered to our guidelines on the number of tables and figures for your article type?<br><br>Data (e.g. quotations) for qualitative studies are not included in the word count, and we prefer that they are integrated into the text (e.g. not in a separate table).	Yes
Study registration	***WHY: Because this means readers understand how you planned your study***<br>Where appropriate have you included details (including reference number, date of registration and URL) of study registration on a database e.g. trials or review database. If your study has a published protocol, is this referenced within the paper?	Reference number and link included
Other study publications?	***WHY: So readers can understand the full context of your study***<br>If there are other publications from this study are these referenced within the body of the paper? Please do not reference papers in preparation or submitted, but in-press publications are acceptable.	N/A
Scales, measures or questionnaires	***WHY: So readers can understand your paper in the context of this information***<br>If your study primarily reports the development or testing of scales/measures or questionnaires have you included a copy of the instrument as a supplementary file?	N/A

For Peer Review

Abbreviations	***WHY: Because abbreviations make a paper hard to read, and are easily misunderstood***<br>Have you removed **all** abbreviations from the text except for extremely well known, standard abbreviations (e.g. SI units), which should be spelt out in full first? We do not allow abbreviations for core concepts such as palliative or end of life care.	Database abbreviations included<br>Other abbreviations outlined and included to enhance the readability of some sentences are:<br>Life-limiting conditions (LLCs)<br>Life-threatening conditions (LTCs)
Research ethics and governance approvals for research involving human subjects	***WHY: We will only publish ethically conducted research, approved by relevant bodies***<br>Have you given full details of ethics/governance/data protection approvals with reference numbers, full name of the committee(s) giving approval and the date of approval? If such approvals are not required have you made it explicit within the paper why they were not required. Are details of consent procedures clear in the paper?	Ethical approval not required
Date(s) of data collection	***WHY: So readers understand the context within which data were collected***<br>Have you given the dates of data collection for your study within the body of your text? If your data are over 5 years old you will need to articulate clearly why they are still relevant and important to current practice.	Yes, and full list of references
Structured discussion	***WHY: So readers can find key information quickly***<br>Papers should have a structured discussion, with sub headings, summarising the main findings, addressing strengths and limitations, articulating what this study adds with reference to existing international literature, and presenting the implications for practice.	Structured discussion guidelines adhered to.
Case reports	***WHY: So that participants are protected, and its importance made clear***<br>If your study is a case report have you followed our clear structure for a case report, including highlighting what research is needed to address the issue raised? Have you made clear what consent was required or given for the publication of the case report? Have you provided evidence of such consent as a supplementary file to the editor?	N/A
Acknowledgements and declarations	***WHY: So readers understand the context of the research***	Acknowledgements and declarations included in manuscript.

For Peer Review

	Have you included a funding declaration according to the SAGE format? Are there acknowledgements to be made? Have you stated where data from the study are deposited and how they may be available to others? Have you conflicts of interest to declare?	
Supplementary data and materials	***WHY: So the context is clear, but the main paper succinct for the reader***<br>Is there any content which could be provided as supplementary data which would appear only in the online version of accepted papers? This could include large tables, full search strategies for reviews, additional data etc.	Two supplementary tables included – table outlining the characteristics of the studies, and a table with examples of data used to inform the CMOCs
References	***WHY: So people can easily find work you have referenced***<br>Are your references provided in SAGE Vancouver style? You can download this style within Endnote and other referencing software.	SAGE Vancouver style references
Ownership of work.	Can you assert that you are submitting your original work, that you have the rights in the work, that you are submitting the work for first publication in the Journal and that it is not being considered for publication elsewhere and has not already been published elsewhere, and that you have obtained and can supply all necessary permissions for the reproduction of any copyright works not owned by you.	This is original work.<br>It is not being considered for publication elsewhere.

For Peer Review

PRISMA 2009 Checklist

Section/topic	#	Checklist item	Reported on page #
**TITLE**			
Title	1	Identify the report as a systematic review, meta-analysis, or both.	1
**ABSTRACT**			
Structured summary	2	Provide a structured summary including, as applicable: background; objectives; data sources; study eligibility criteria, participants, and interventions; study appraisal and synthesis methods; results; limitations; conclusions and implications of key findings; systematic review registration number.	2
**INTRODUCTION**			
Rationale	3	Describe the rationale for the review in the context of what is already known.	4
Objectives	4	Provide an explicit statement of questions being addressed with reference to participants, interventions, comparisons, outcomes, and study design (PICOS).	5
**METHODS**			
Protocol and registration	5	Indicate if a review protocol exists, if and where it can be accessed (e.g., Web address), and, if available, provide registration information including registration number.	5
Eligibility criteria	6	Specify study characteristics (e.g., PICOS, length of follow-up) and report characteristics (e.g., years considered, language, publication status) used as criteria for eligibility, giving rationale.	7
Information sources	7	Describe all information sources (e.g., databases with dates of coverage, contact with study authors to identify additional studies) in the search and date last searched.	6,9
Search	8	Present full electronic search strategy for at least one database, including any limits used, such that it could be repeated.	9
Study selection	9	State the process for selecting studies (i.e., screening, eligibility, included in systematic review, and, if applicable, included in the meta-analysis).	11
Data collection process	10	Describe method of data extraction from reports (e.g., piloted forms, independently, in duplicate) and any processes for obtaining and confirming data from investigators.	7,8
Data items	11	List and define all variables for which data were sought (e.g., PICOS, funding sources) and any assumptions and simplifications made.	7,8
Risk of bias in individual studies	12	Describe methods used for assessing risk of bias of individual studies (including specification of whether this was done at the study or outcome level), and how this information is to be used in any data synthesis.	7
Summary measures	13	State the principal summary measures (e.g., risk ratio, difference in means).	n/a
Synthesis of results	14	Describe the methods of handling data and combining results of studies, if done, including measures of consistency (e.g., I^2) for each meta-analysis.	7,8

For Peer Review

PRISMA 2009 Checklist

Section/topic	#	Checklist item	Reported on page #
Risk of bias across studies	15	Specify any assessment of risk of bias that may affect the cumulative evidence (e.g., publication bias, selective reporting within studies).	20,21
Additional analyses	16	Describe methods of additional analyses (e.g., sensitivity or subgroup analyses, meta-regression), if done, indicating which were pre-specified.	n/a
**RESULTS**			
Study selection	17	Give numbers of studies screened, assessed for eligibility, and included in the review, with reasons for exclusions at each stage, ideally with a flow diagram.	9-11
Study characteristics	18	For each study, present characteristics for which data were extracted (e.g., study size, PICOS, follow-up period) and provide the citations.	7 (and appendix 1)
Risk of bias within studies	19	Present data on risk of bias of each study and, if available, any outcome level assessment (see item 12).	n/a
Results of individual studies	20	For all outcomes considered (benefits or harms), present, for each study: (a) simple summary data for each intervention group (b) effect estimates and confidence intervals, ideally with a forest plot.	n/a
Synthesis of results	21	Present results of each meta-analysis done, including confidence intervals and measures of consistency.	n/a
Risk of bias across studies	22	Present results of any assessment of risk of bias across studies (see Item 15).	20,21
Additional analysis	23	Give results of additional analyses, if done (e.g., sensitivity or subgroup analyses, meta-regression [see Item 16]).	n/a
**DISCUSSION**			
Summary of evidence	24	Summarize the main findings including the strength of evidence for each main outcome; consider their relevance to key groups (e.g., healthcare providers, users, and policy makers).	20
Limitations	25	Discuss limitations at study and outcome level (e.g., risk of bias), and at review-level (e.g., incomplete retrieval of identified research, reporting bias).	20,21
Conclusions	26	Provide a general interpretation of the results in the context of other evidence, and implications for future research.	21,22
**FUNDING**			
Funding	27	Describe sources of funding for the systematic review and other support (e.g., supply of data); role of funders for the systematic review.	1

From: Moher D, Liberati A, Tetzlaff J, Altman DG, The PRISMA Group (2009). Preferred Reporting Items for Systematic Reviews and Meta-Analyses: The PRISMA Statement. PLoS Med 6(6): e1000097. doi:10.1371/journal.pmed1000097

For more information, visit: **www.prisma-statement.org**.

For Peer Review
